# Remediating cognitive inflexibility in obsessive compulsive disorder and anorexia nervosa neither moderates nor mediates treatment effects: an exploratory study

**DOI:** 10.3389/fpsyt.2024.1456890

**Published:** 2025-01-13

**Authors:** Boris van Passel, Unna N. Danner, Alexandra E. Dingemans, Theo G. Broekman, Lot C. Sternheim, Eni S. Becker, Annemarie A. van Elburg, Eric F. van Furth, Gert-Jan Hendriks, Daniëlle C. Cath

**Affiliations:** ^1^ Overwaal Centre of Expertise for Anxiety Disorders, Obsessive Compulsive Disorder (OCD) and Posttraumatic Stress-Disorder (PTSD), Institution for Integrated Mental Health Care, Pro Persona, Nijmegen, Netherlands; ^2^ Behavioural Science Institute, Radboud University, Nijmegen, Netherlands; ^3^ Altrecht Eating Disorders Rintveld, Zeist, Netherlands; ^4^ Department of Clinical Psychology, Utrecht University, Utrecht, Netherlands; ^5^ Rivierduinen Eating Disorders Ursula, Leiden, Netherlands; ^6^ Bureau Bêta, Nijmegen, Netherlands; ^7^ Department of Psychiatry, Leiden University Medical Center, Leiden, Netherlands; ^8^ Radboud University Medical Centre, Department of Psychiatry, Radboud University, Nijmegen, Netherlands; ^9^ Department of Specialist Training, Institution for Integrated Mental Health Care (GGz) Drenthe, Assen, Netherlands; ^10^ Department of Psychiatry, University of Groningen & University Medical Center Groningen, Groningen, Netherlands

**Keywords:** obsessive compulsive disorder, anorexia nervosa, cognitive flexibility, cognitive remediation therapy, moderation analysis

## Abstract

**Objective:**

Obsessive compulsive disorder (OCD) and anorexia nervosa (AN) are conditions associated with poor cognitive flexibility, a factor considered to interfere with treatment, but research into the relationship between cognitive flexibility and treatment outcome is limited. This study explores whether baseline measures of cognitive flexibility predict outcomes in OCD and AN, evaluates whether changes in these measures contribute to treatment outcome, and evaluates the effectiveness of adjunctive cognitive remediation therapy (CRT) in improving cognitive flexibility.

**Methods:**

This secondary analysis utilized linear mixed model analysis on data from a randomized controlled multicenter clinical trial involving adult participants with OCD (n=71) AND AN (n=61). Participants underwent 10 twice-weekly sessions of either CRT or a non-specific active control intervention (specialized attention therapy; SAT), followed by treatment as usual. Assessments using Yale-Brown Obsessive Compulsive Scale and the Eating Disorder Examination Questionnaire were conducted at baseline, post-CRT/SAT and at 6 and 12 months. Cognitive flexibility was evaluated through the Trail Making Test (TMT), the Color-Word Interference Test (CWIT) and the Detail and Flexibility Questionnaire (DFlex).

**Results:**

Levels of cognitive flexibility at baseline did not predict or moderate treatment outcome, nor did change in cognitive flexibility (baseline post-CRT/SAT) mediate treatment outcome, with CRT providing no greater improvement in measures of cognitive flexibility than SAT.

**Conclusions:**

This study failed to find any relationship between measures of cognitive flexibility and treatment outcome in OCD and AN, and thus questions hypothetical associations between measures of cognitive flexibility and mechanisms of change in patients with OCD and AN.

## Introduction

Obsessive-compulsive disorder (OCD) and anorexia nervosa (AN) are severe mental disorders with extensive impact on psychological well-being. Some authors have suggested that OCD and AN belong to the same spectrum of disorders (1, 2), basing their postulations on five similarities. First, these disorders share clinical features, including obsessive worrying, compulsive and ritualistic behavior, and repetitive thinking (3). Second, the presence of OCD has been hypothesized to be a predisposing factor for the development of AN (4); third, OCD frequencies in AN are significantly elevated, ranging between 19% in cross-sectional studies and 44% in longitudinal studies (5). Further, AN is diagnosed in increased frequencies up to 10% in women diagnosed with OCD (6). Fourth, there is a high genetic correlation (*r_g_* =0.49 ± 0.13, *p*<0.01) between AN and OCD (7), and, finally, individuals with AN and OCD have been shown to share inefficiencies in executive functioning (8). There is growing evidence that cognitive *in*flexibility represents a core feature of the neuropsychological profiles of both OCD and AN (9–11) and entails a candidate neuropsychological endophenotype, i.e. a set of stable behavioral symptoms with a clear genetic connection (12–16). In a recent systematic review of publications on cognitive flexibility in acute AN, Miles et al. (17) found that adult participants with AN perform worse than healthy controls (HCs) on neuropsychological indices and self-report items of cognitive flexibility.

Executive functions refer to the collection of cognitive processes necessary for the cognitive control of goal-directed behavior (18) and include ‘core’ functions such as cognitive flexibility, inhibitory control, working memory as well as the higher-level functions of reasoning, problem-solving, and planning (19). In both OCD and AN, specifically cognitive flexibility has been found to be impaired (17, 20, 21), which function is typically defined as the ability to change perspectives or approaches to a problem and readily adjust to new demands, rules, or priorities (19). When changes take place in the environment, we need to be able to focus our attention on those elements that are changing and, after discovering that a previous approach does no longer apply to the changed environment, we are expected to be able to suppress our earlier response and develop a new strategy. We can thus integrate information and manipulate it in real time to flexibly switch from one response scenario to another (22).

An essential part of cognitive flexibility is set-shifting, the ability to move back and forth between tasks, operations, or mental sets in response to changing goals or environmental experiences (23), which has been suggested to be inhibited in both OCD (24) and AN (25). Snyder et al. (10) found their OCD group to perform worse than the HC group on the Trail Making Test (TMT), with the between-group differences showing medium effect sizes (*d*=0.54); the Wisconsin Card Sorting Task (WCST) and the Intra-Extra-dimensional Set-shift Task (ID/EDS) yielded both smaller, comparable effect sizes (*d*=0.44 and *d*=.50, respectively). A meta-analysis of OCD studies confirmed impaired set-shifting performance on the ID/EDS, with medium-to-large effect sizes, which impairment was also found to extend to the participants’ clinically asymptomatic first-degree relatives (26, 27). Another meta-analysis evaluating 11 studies with participants with the AN restricting subtype reported medium effect sizes (*g*=-0.51) for inefficient set-shifting (25).

Set-shifting deficits in OCD and AN have been found to be mediated by abnormal activation of fronto-striatal circuitry, areas that are important for executive functions (e.g. dorsolateral/ventrolateral prefrontal and striatal regions) (28–30). Findings revealed that patients with OCD made more errors and lacked activation in the dorsal fronto-striatal regions linked to cognitive flexibility, suggesting that diminished cognitive flexibility contributes to their task deficits. Interestingly, fronto-striatal dysfunction in OCD is amenable to treatment (31). Similarly, in AN, altered activation in fronto-striatal regions and limbic circuits are taken to mediate the development of the disorder (32, 33). In AN, neural mechanisms that define the therapeutic response to CBT are currently being studied but not yet elucidated. Similarly, individuals with AN struggle with flexible behavior adaptation, marked by reduced activity in fronto-striatal circuits associated with behavioral changes (29, 34).

Together, the findings described demonstrate that adults with OCD and those with AN score worse than HCs on neuropsychological as well as subjective measures of cognitive flexibility, including set-shifting. The literature on the association between reduced cognitive flexibility and symptom severity is contradictory. Some studies have found this association, e.g. (35, 36), while others have not, e.g. (37, 38). These mixed findings are likely related to the different methods used to measure cognitive flexibility and the various outcome measures employed (e.g., BMI vs. EDE-Q). Findings revealed that patients with OCD made more errors and lacked activation in the dorsal fronto-striatal regions linked to cognitive flexibility, suggesting that diminished cognitive flexibility contributes to their task deficits. Similarly, individuals with AN struggle with flexible behavior adaptation, marked by reduced activity in fronto-striatal circuits associated with behavioral changes (29, 34).

In addition to the above-mentioned neuropsychological measures, the detail and flexibility questionnaire (DFlex) (39) revealed significantly poorer subjective cognitive flexibility in adolescents and adults with AN when compared to HCs (39–41), while, again compared to HCs, female students with subclinical obsessive-compulsive symptoms likewise showed significantly more self-reported cognitive inflexibility (42). Finally, recently we reported on the baseline neuropsychological and subjective measures of cognitive flexibility using the same patient groups as reported here directly comparing participants with OCD and AN to HCs and found both patient groups to show similar results with inflexibility, where the higher rates of *perceived* inflexibility did not correlate with the neuropsychological cognitive flexibility scores (43).

Although the identified correlations between cognitive inflexibility and symptom severity do not imply a causal relationship, one hypothesis could be that interventions targeting the enhancement of cognitive flexibility may result in greater symptom reduction and a larger therapeutic effect in both populations.

Cognitive remediation therapy (CRT) is an easy-to-use intervention designed to do just that for people coping with AN (44). AN case series, uncontrolled studies and RCTs had yielded promising results (45–51). For OCD, CRT had not been previously investigated as a treatment enhancer until our study (52). However, there were two studies demonstrating that cognitive training was effective in improving cognitive flexibility, with one study also showing a positive effect on reducing symptom severity (53, 54). On two other studies (55, 56) involving patients with OCD, no significant differences were found between the effects of cognitive training and a non-cognitive training control condition on neuropsychological measures and a symptom-specific outcome measure. Moreover, recent randomized controlled trials (RCTs) and a preliminary systematic review and meta-analysis predominantly reported negative results or non-superiority compared to other control treatments when CRT was used as an enhancer to treatment as usual for eating disorders and OCD (57–62). This suggests that while poor cognitive flexibility may interfere with OCD and AN treatment, CRT has yielded less favorable outcomes than anticipated.

While CRT is assumed to improve cognitive flexibility and central coherence, this premise remains debated. Furthermore, RCTs of CRT with control groups are scarce, particularly those examining neuropsychological outcomes. To address this gap, we conducted an overview of the evidence on the effects of CRT or similar cognitive training interventions on neuropsychological measures in individuals with anorexia nervosa or OCD.

A search of PsycINFO and PubMed, using the terms ((OCD or Obsessive Compulsive Disorder) or (Anore*)) and (cognit* and (remed* or train*)) and (Neuropsychol* or measure), yielded 277 unique results. We excluded studies focused on children or adolescents, as well as those lacking a control condition for comparison. Relevant articles identified through citation tracking were added. This process produced a final selection of 15 studies comparing cognitive training with a control condition or waitlist, summarized in **Table 1**.

**Table 1:** Effectiveness of cognitive remediation therapy compared to control interventions on neuropsychologic measures and/or symptom severity.

Study	Design	Sample size	Diagnosis	Outcome measures	Between group difference	Favors C(R)T (Y/N)
Park et al. (53)	CT vs. control	*n*=30	OCD	Y-BOCS RCFT K-CVLT	*p*<0.05 *p*<0.05 *p*<0.05	Y Y Y
Buhlmann et al., (54)	CT vs. no training	*n*=35	OCD	RCFT	*p*<0.001	Y
Jelinek et al., (55)	CT vs. non CT	*n*=21	OCD	RIF	n.s.	N
Davies et al., (63)	CT vs. non CT	*n*=81	AN	Brixton WCST Frag Pic GEFT Stroop Angry Stroop Social RME	n.s. n.s. n.s. n.s. n.s. n.s. n.s.	N N N N N N N
Lock et al. (64)	CRT+CBT vs. CBT only	*n*=46	AN	CWIT RCFT BMI EDE	*p=*0.006^*^ *p*=0.013^*^ n.s. n.s.	Y Y N N
Calkins and Otto, (56)	CT vs. PVT	*n*=48	OCD	OCI-r Anagram-task PASAT performance	n.s. n.s. n.s.	N N N
Brockmeyer et al. (49)	CRT+TAU vs. NNT+TAU	*n*=40	AN	Cued Task-switching paradigm	*p*=0.027	Y
Garret et al., (48)	CRT+CBT vs. CBT only	*n*=21	AN	WCST	*p*=0.03	Y
Dingemans et al. (47)	CRT+TAU vs. TAU only	*n* =82	AN / EDNOS-AN / BN	EDE-Q BMI WCST errors TMT RCFT	*p*<0.05 n.s. n.s. n.s. n.s.	Y N N N N
Lock et al. (62)	CRT+FBT vs. AT+FBT	*n*=30	AN	EDE BMI WCST RCFT	*p=*0.03^**^ n.s. n.s. n.s.	N N N N
Sproch et al. (60)	CRT+TAU vs. TAU only	*n*=275	AN	WCST TMT	n.s. n.s.	N N
Cameron et al. (65)	Goal Management Training vs. Waiting List	*n*=19	OCD	Y-BOCS CPT Stroop TOL CVLT CFQ DEX MACCS	n.s. *p*=0.06 n.s. *p*=0.47 n.s. *p*=0.07 n.s. n.s.	N Y N Y N Y N N
van Passel et al. (58)	CRT+TAU vs. SAT+TAU	*n*=71 *n*=61	OCD AN	Y-BOCS EDE-Q DFlex	p<0.01^**^ n.s. n.s.	N N N
Brockmeyer et al., (57)	CRT+TAU vs. ART+TAU	*n*=167	AN	EDE-Q BMI WCST TMT NT DFlex	n.s. n.s. n.s. *p*=0.018 n.s. n.s.	N N N N N N
Meneguzzo et al. (51)	CRT + inpatient treatment vs. TAU + inpatient treatment	*n*=59	AN	CFS OCI-R RCS DFlex CFI	*p*=0.050 *p*=0.013 *p<0.001* *p*=0.005 *p*=0.001	Y Y Y Y Y

ACI, Attention control intervention; AS, Association splitting; AVLT, Auditory Verbal Learning test; CFI, Cognitive Flexibility Inventory; CFQ, Cognitive Failures Questionnaire; CFS, Coping Flexibility Scale; CPT, Conners’ Continuous Performance Task; (c)CRT, (computerized) Cognitive Remediation Therapy; CT, Cognitive training; DEX, Dysexecutive Questionnaire; DFlex, detail and flexibility questionnaire; Frag Pic, Fragmented Pictures Task; GEFT, Group Embedded Figures Task; K-CVLT, Korean-California Verbal Learning Test; MACC, Memory and Cognitive Confidence Scale; NT, Navon task; PASAT: Paced Auditory Serial Addition Task; PDQ, Perceived deficits questionnaire; PVT, Peripheral vision task; RCS, Resistance to Change Scale; RCFT, Rey-Osterrieth Complex Figure Test; RIF, Retrieval-induced forgetting paradigm; RME, Reading the Mind in the Eyes Task; SAT, Specialized attention training; SDS, Sheehan Disability Scale; Stroop Angry, Pictorial Emotional Stroop Task, angry faces condition; Stroop social, Pictoral Emotional Stroop Task, social stimuli condition; TAU, Treatment as usual; TMT, Trail making test; TOL, Tower of London; WCST, Wisconsin card sorting task.

^*^ significant group difference at session 8, but not at the end of treatment (*p*=0.143, and *p*=0.395 respectively).

^**^ significant difference in favor of control therapy.n.s., not significant.

**Table 1** presents studies with CRT, Cognitive Remediation and Emotion Skills training (CREST) or comparable cognitive training focused on cognitive inflexibility in adults with AN or OCD to control treatments. The table shows the effect on symptom reduction and neuropsychological measures. As can be seen, the impact of these cognitive trainings on symptom reduction and neuropsychological measures is mixed. While the evidence summarized in **Table 1** highlights the variability in outcomes of cognitive training interventions, it underscores the need for further investigation into their specific effects. To address this, our study aims to explore three key questions: a) to what extend does CRT lead to greater improvements on neuropsychological and subjective measures of cognitive flexibility as compared to Specialized attention therapy (SAT), our custom-designed active control condition, and b) to what extend does cognitive flexibility measured at baseline predict treatment outcomes in OCD and AN, and c) do changes in cognitive flexibility contribute to the treatment outcomes.

## Methods

### Participants

The participants being evaluated in this study (n=132) were originally enrolled in our RCT evaluating the effectiveness of CRT versus SAT as enhancers of TAU for OCD or AN (52, 58). Participants were between 18 and 66 years old and fulfilled the DSM-IV-TR^1^ criteria for OCD (*n*=71) or AN or eating disorder not otherwise specified-AN type (EDNOS-AN, *n*=61). To verify the DSM-IV-TR diagnoses we used the Structured Clinical Interview for DSM-IV-TR axis-I disorders (66). We included participants with AN and EDNOS-AN because meta-analyses (67, 68) concluded that these conditions fall within the same spectrum in terms of eating pathology, general psychopathology and physical health. Of all participants with AN or EDNOS-AN, (3.3%, *n*=3) had a BMI ≥ 18.5. AN diagnoses were additionally confirmed using the ED examination (EDE) interview (69) or the self-report questionnaire (EDE-Q) (70). Participants with OCD were included when they scored ≥ 16 on the Yale-Brown obsessive-compulsive scale (Y-BOCS). Comorbid OCD and AN was allowed. Twelve participants fulfilled the diagnostic criteria for both OCD and AN.

Exclusion criteria were severe neurological illness (including a history of seizures, stroke, or Parkinson’s disease), severe comorbid mental disorders (e.g. schizophrenia, clinically significant bipolar disorder, current psychosis, substance dependence/abuse, organic mental disorder), intellectual impairment [defined as an IQ<80 as estimated with the Dutch Adult Reading Test (DART)] (71), and an inability to adequately speak or read Dutch. Antidepressants and antipsychotics were allowed, if dosages were kept constant during the experimental part of the study. Since benzodiazepines can dampen the effect of cognitive treatments (72), only sleep medication was allowed, restricted to a daily dose of up to 20 mg of temazepam (or equivalent dosages of other sleep aids). Participants did not receive renumeration for their participation.

The demographic and clinical characteristics of the study participants are provided in **Table 2**; detailed descriptions have been published elsewhere (43, 58). Of the 61 participants with AN enrolled, 57 were female (93.4%) and 4 male (6.6%); mean age was 24.90 years (range 18-52, SD 7.28). Of the 72 participants with OCD that were included, 50 were female (69.4%) and 22 male (30.6%) and their mean age was 33.92 years (range 18-66, SD 10.86). Participants were randomized to CRT (*n*=68) or SAT (*n*=64). One male participant with OCD withdrew his consent just before randomization and the baseline assessment. The two intervention groups showed no significant differences in self-reported sex/gender, level of education, number of previous treatments, illness duration, or severity of the illness as based on the EDE-Q (AN) and the Y-BOCS scores (OCD). At baseline, there were no differences in the neuropsychological and self-report flexibility indices between the groups receiving CRT and SAT, respectively.

**Table 2:** Mean, SD and between group differences for demographic, clinical and flexibility variables of all groups.

Characteristic	Overall *n* = 132*^1^*	CRT *n* = 68*^1^*	SAT *n* = 64*^1^*	CRT vs. SAT
**Diagnosis: Main diagnosis**				χ^2^(1)=0.00, *p*>0.9
**AN**	61 (46%)	31 (46%)	30 (47%)	
** OCD**	71 (54%)	37 (54%)	34 (53%)	
**Gender:**				χ^2^(1)=0.38, *p*=0.5
** male**	25 (19%)	11 (16%)	14 (22%)	
** female**	107 (81%)	57 (84%)	50 (78%)	
**Years of education**	8.63 (1.93)	8.52 (1.95)	8.77 (1.92)	*t*=-0.64, *p*=0.5
** Unknown**	31	14	17	
**Years of education, discrete**				χ^2^(6)=3.9, *p*=0.7
** 4**	6 (5.9%)	3 (5.6%)	3 (6.4%)	
** 5**	5 (5.0%)	3 (5.6%)	2 (4.3%)	
** 6**	3 (3.0%)	3 (5.6%)	0 (0%)	
** 8**	29 (29%)	15 (28%)	14 (30%)	
** 9**	32 (32%)	17 (31%)	15 (32%)	
** 10**	1 (1.0%)	1 (1.9%)	0 (0%)	
** 11**	25 (25%)	12 (22%)	13 (28%)	
** Unknown**	31	14	17	
**DART-score**	83.06 (12.81)	84.11 (10.67)	81.93 (14.77)	*t*=0.94, *p*=0.4
** Unknown**	8	4	4	
**PreviousTreatment: Number of psychological treatments main diagnosis**				χ^2^(3)=0.31, *p*>0.9
** 0**	37 (31%)	20 (33%)	17 (30%)	
** 1 to 5**	44 (37%)	23 (38%)	21 (37%)	
** 5 to 10**	9 (7.6%)	4 (6.6%)	5 (8.8%)	
** More than 10**	28 (24%)	14 (23%)	14 (25%)	
** Unknown**	14	7	7	
**Duration from first diagnosis**	6.75 (9.08)	6.31 (8.28)	7.22 (9.91)	*t*=-0.55, *p*=0.6
** Unknown**	12	6	6	
**EDE-Q**	4.05 (1.26)	4.00 (1.31)	4.12 (1.23)	*t*=-0.34, *p*=0.7
** Unknown**	78	39	39	
**YBOCS**	24.09 (6.46)	23.81 (7.15)	24.41 (5.68)	*t*=-0.39, *p*=0.7
** Unknown**	64	32	32	
**TMT AB-ratio**	1.52 (0.42)	1.47 (0.40)	1.58 (0.43)	t=-1.5, p=0.14
** Unknown**	8	2	6	
**CWIT-Rigidity**	57.48 (20.86)	55.93 (13.98)	59.24 (26.62)	*t*=-0.87, *p*=0.4
** Unknown**	4	0	4	
**DFlex-Rigidity**	43.98 (12.06)	43.98 (12.75)	43.96 (11.35)	*t*=0.01, *p*>0.9
** Unknown**	11	4	7	

*^1^* n (%); Mean (SD).

*^2^* Pearson's Chi-squared test; Welch Two Sample t-test.

DART, Dutch Adult Reading Test; EDE-Q, Eating Disorder Examination-Questionnaire; Y-BOCS, Yale-Brown Obsessive-Compulsive Scale; TMT AB ratio, Trail Making Test, ratio part A: part B; CWIT-Rigidity, Color Word Interference Test, Rigidity subscale; DFlex-Rigidity, Detail and Flexibility Questionnaire, Rigidity subscale; CRT, Cognitive Remediation Therapy; SAT, Specialized Attention Therapy; AN, Anorexia Nervosa; OCD, Obsessive-Compulsive disorder.Bold values indicate significant values.

### Procedure

Participants were originally enrolled in our RCT evaluating the effectiveness of CRT versus SAT as enhancers of TAU for OCD or AN (52, 58) of which details have been described elsewhere. In brief, the study was conducted at four highly specialized OCD and AN treatment-centers in The Netherlands. Participants gave informed consent prior to enrollment and were 1:1 randomized to one of two arms (CRT or SAT). CRT, based on the manual of Tchanturia (73) aimed to enhance cognitive flexibility and reduce over-detailed thinking through ten 45-minute biweekly sessions with reflective tasks and homework. The control condition that we designed for this trial, named Specialized Attention Treatment (SAT), was equal to CRT with respect to duration, homework assignments and timing but focused solely on neutral relaxing entertainment and experiences (e.g., board games, listening music, looking at a photo album) without targeting cognitive flexibility or thinking styles (74). After 10 sessions of CRT or SAT, all participants received TAU for OCD or AN, following Dutch and international guidelines (75–78) including CBT with exposure, psychoeducation, cognitive therapy, and pharmacotherapy for OCD, and comprehensive care for AN, such as CBT based protocols, art therapy, social skills training, family therapy, and pharmacotherapy.

### Measures

Participants completed all measures described below at baseline (T0), directly after CRT/SAT (T1), and 6 (T2) and 12 (T3) months after baseline.

#### Symptom severity measures

The Eating Disorder Examination Questionnaire (EDE-Q) (70, 79) is the self-report version of the Eating Disorder Examination (EDE) (80), a semi-structured interview to evaluate ED psychopathology. The EDE-Q assesses attitudinal and behavioral aspects of EDs over a 28-day period using four subscales gauging concerns about shape, weight and eating, and restraint, generating subscale scores and a total scale score. The EDE-Q has excellent internal consistency (Cronbach α 0.78-0.93). The subscales have excellent test-retest reliability over a 2-week period (Pearson’s r ranging from 0.81 to 0.94) (81).

The 10-item Yale-Brown Obsessive-Compulsive Severity Scale (Y-BOCS) (82) is a clinician-rated, semi-structured interview-based scale that is broadly used to assess obsessive-compulsive symptom severity. The scale has two parts, with each 5-item subscale examining five aspects of OCD pathology: 1) time consumed, 2) degree of interference, 3) distress, 4) resistance, and 5) perceived control. The first subscale gives an obsession score (maximum: 20), the second a compulsion score (maximum: 20), together yielding a total score (maximum: 40). The Y-BOCS has a strong internal consistency (Cronbach α.88-.91), inter-rater reliability (r 0.82-0.98), and test-retest reliability in clinical and nonclinical samples was excellent.

#### Measures of cognitive flexibility

##### Neuropsychological measures

The neuropsychological measures we used comprised the Trail Making Test (TMT) (83), and the Stroop task (84), including Delis-Kaplan Executive Function System (D-KEFS) card nr. 4 (85).

The TMT (83) was administered to evaluate set-shifting abilities. Originally a pen-and-paper test, we used the computerized version that has recently become available. Patients numerically or alphabetically connect circles on a page in a ‘dot-to-dot’ fashion (trail A), and then alternatively link numbers and letters, i.e. 1–A–2–B–3–C (trail B). The AB ratio score, i.e. the ratio between the time taken to complete trail A and the time needed to complete part B, serves as the index of cognitive flexibility.

The Color-Word Interference Test (CWIT), part of the D-KEFS test battery (85), comprises four components: color naming, word reading, inhibition, and inhibition/switching. In the first part, participants are required to quickly and accurately name color patches. In the second part, participants read out words printed in black ink. The third part involves an inhibition task, where participants identify the ink colors of color words printed in incongruous colors. Lastly, the test assesses the ability to switch between cognitive tasks without explicit cues. Participants are instructed to name the color of the ink when seeing words, but if a word appears within a box, they are instructed to read the word. These boxes are randomly positioned throughout the trial. The time taken to complete the fourth CWIT card is used as the measure of cognitive flexibility. The D-KEFS CWIT has an internal consistency of between 0.72 and.82 and a test-retest reliability of 0.65 for condition 4 (85).

##### Self-report questionnaire

The DFlex (39) is a self-report scale that measures cognitive rigidity and attention to detail (central coherence). Patients are asked to rate 24 statements on 6-point Likert scales with anchors ‘strongly agree’ and ‘strongly disagree’. The two subscales showed excellent internal consistency (Cronbach α 0.90 and 0.91, respectively). Construct validity (as compared to relevant subscales of the autism-spectrum quotient (AQ) (86) was strong for cognitive rigidity (r = 0.72) but only moderate for attention to detail (r = 0.26) (39).

### Statistical analyses

Prior to analysis, variables were evaluated for the presence of outliers and distributional properties were examined. We determined outliers in a univariate way when the measurements exceeded the 5% or 95% threshold from the sample. These measurements were excluded from analyzes. Data analyses were conducted with R version 4.1.1 (87) using R Studio 2021.09.0 + 351. Longitudinal modelling was performed using the R lme4 package (88) and the exceedance probabilities (*p* values) of the parameters were calculated with the R package lmertest (89). Between-group differences were analyzed using **χ^2^** for categorical variables and independent sample t-tests for continuous variables.

To analyze change in cognitive flexibility from T0 to T1, we specified a linear mixed model for each of the flexibility indices, with the measure of flexibility as the dependent variable and as fixed effects: the actual day of measurement (time), diagnosis (AN/OCD), CRT/SAT, baseline flexibility score, and the interactions between diagnosis and time, between CRT/SAT and time, and between diagnosis, CRT/SAT and time. In the random effects part of the model, random slopes for the time effect were included. Estimated marginal means and within-group differences were calculated for baseline and T1 scores using the emmeans package (90). We included all participants with at least two measurements. From the original sample (n=132; 58 CRT and 47 SAT), 27 patients were excluded (10 from the CRT group and 17 from the SAT group) due to being classified as outliers or having only a single measurement. To specifically address missing data in longitudinal analyzes, we utilized Linear Mixed Models. This approach is more robust in handling missing data as it considers the correlations between measurements from the same participants over time, estimating parameters using all available data and without imputation of missing data.

For the moderation and mediation analyses, linear mixed models were fitted with severity (the z-score from the Y-BOCS for OCD and the z-score from the EDE-Q for AN) as the dependent variable. We specified a linear mixed model with fixed effects: baseline flexibility score as the moderator, or - for the mediation analyses - the baseline-to-T1 difference score for the measures of cognitive flexibility as a mediator, the interaction between moderator (or mediator) and time. We did not include effects for diagnosis or type of adjunctive treatment (CRT or SAT) in this model because there was no significant interaction between time and diagnosis or time and type of adjunctive treatment in the flexibility change models; in our earlier study with the same sample (58), we examined the effects of pharmacotherapy on treatment response and found that differences in psychotropic medication were non-significant and did not confound treatment effects; therefore medication use was not controlled for. To account for the two different phases, we specified a linear B-spline model with a knot at the end of CRT/SAT (knot at T1, degree=1). In the random effects part of the model, random slopes for the time effect were included. To visualize the interaction between the measure of flexibility and flexibility difference score, and severity, we plotted three splines with confidence intervals for three levels of flexibility scores.

## Results

### Does CRT lead to greater improvements on cognitive flexibility as compared to SAT?

The mixed model analyses revealed no significant change from T0 to T1 for TMT AB-ratio (*F*(195)=2.07, *p*=0.152). CWIT-rigidity (*F*(201)=23.0, *p*<0.001) and DFlex-rigidity (*F*(191)=8.38, *p*<0.01) did show a significant T0-T1 change, which implies that flexibility as measured with the CWIT and DFlex rigidity subscales had improved following CRT/SAT. However, the two-way interactions time*diagnosis and time*type of adjunctive treatment were all non-significant. The three-way interaction time*diagnosis*type of adjunctive treatment was also non-significant, signifying there were no differences in change over time between the CRT and SAT or the OCD and AN groups.

As can be seen in **Table 3**, the within-group differences for estimated marginal means (EMMs) as based on the model demonstrate significant improvement over time for the CWIT and DFlex but not for TMT AB-ratio in the total study sample. When calculating EMMs for the diagnosis (AN/OCD) and type of adjunctive treatment (CRT/SAT) subgroups, we found that on the CWIT all subgroups showed significant decreases, reflecting an improvement in cognitive flexibility over time. On the DFlex, only the OCD subgroup showed a significant decrease over time. Finally, we saw no significant T0-to-T1 changes in the TMT AB-ratio scores for any of the subgroups.

**Table 3:** Within-group estimated marginal means (EMMs) for the cognitive flexibility measurements at baseline and 6 weeks for the total group and for the four subgroups.

	Within-group differences in EMMs								
	TMT AB-ratio			CWIT-rigidity			DFlex-rigidity		
	Baseline	T1 (6 weeks)	*p*	Baseline	T1 (6 weeks)	*p*	Baseline	T1 (6 weeks)	*p*
Total Group	1.50	1.46	*0.16*	54.71	50.88	0.00^*^	43.19	40.98	0.00^*^
Subgroups:CRT	1.48	1.49	0.99	54.87	51.18	0.00^*^	43.15	41.36	0.29
SAT	1.53	1.43	0.14	54.56	50.57	0.01^*^	43.23	40.61	0.11
AN	1.51	1.46	0.71	53.82	50.13	0.01^*^	43.16	41.90	0.60
OCD	1.50	1.46	0.79	55.61	51.62	0.01^*^	43.22	40.07	0.04^*^

TMT AB-ratio, Trail Making Test, ratio part A: part B; CWIT-rigidity, Colour-Word Interference Test-rigidity subscale; DFlex-rigidity, Detail and Flexibility Questionnaire-rigidity subscale; CRT, Cognitive Remediation Therapy; SAT, Specialized Attention Therapy; AN, Anorexia nervosa; OCD, Obsessive-compulsive disorder; ^*^ *p*<0.05.Bold values indicate significant values.

### Does cognitive flexibility measured at baseline predict treatment outcomes in OCD and AN?

**Figure 1:**
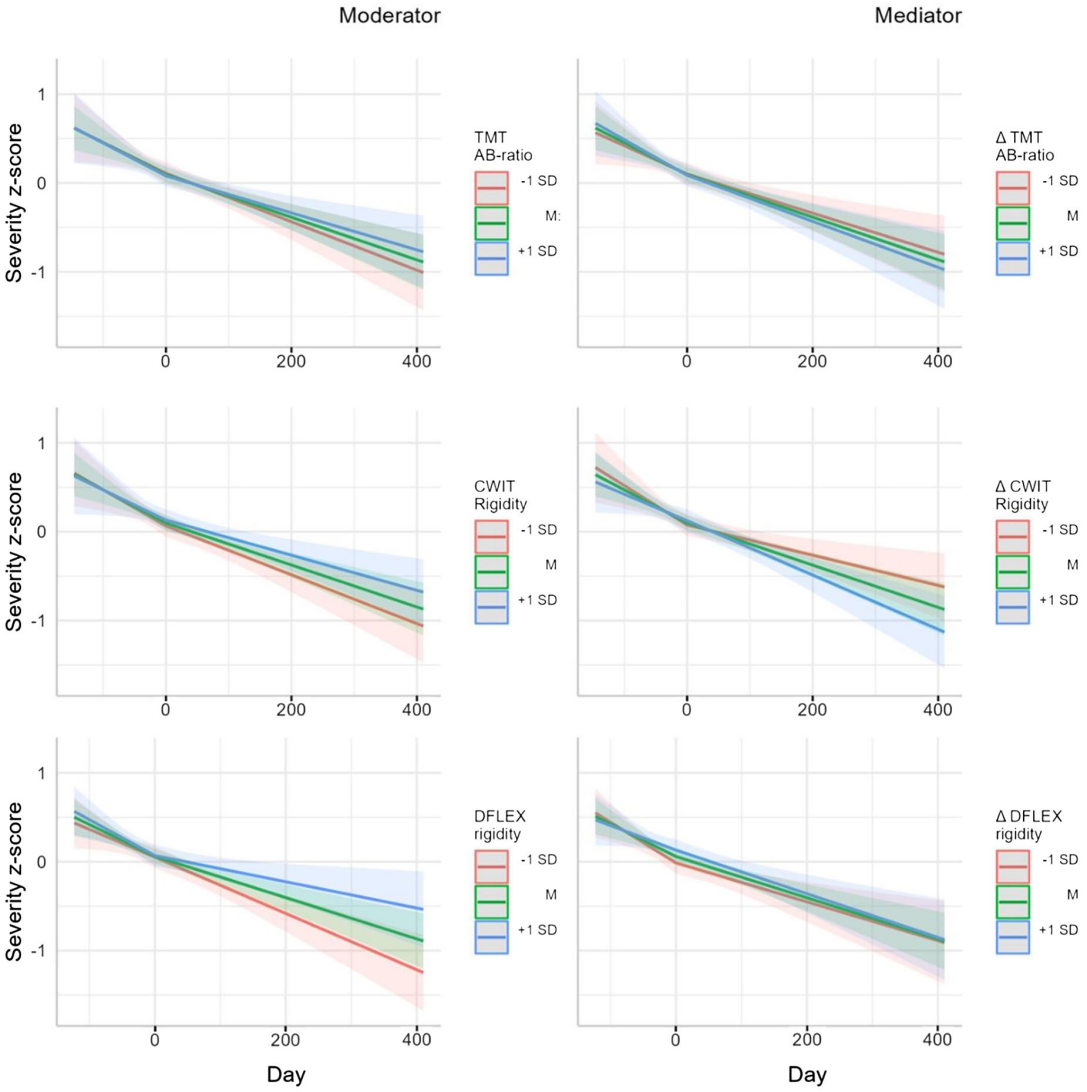
Moderator (left column) and mediator (right column) models: predeicted severity z-score with measurement of flexibility (MF) or MF as covariate.

Presenting the moderator models, **Figure 1** (left column) shows there was no significant interaction between the baseline TMT AB-ratio score and time (*t*(288)=-0.0979, *p*=0.922) during CRT/SAT or the TAU phase (*t*(148)=0.540, *p*=0.590), which tells us that TMT performance did not moderate change in symptom severity during treatment. During CRT/SAT, the interaction between the baseline CWIT-rigidity scores and time was non-significant (t(296)=-0.958, *p*=0.339), which also applies to TAU (t(163)=-2.54, *p*=0.012), indicating that CWIT-rigidity did not moderate changes in Y-BOCS and EDE-Q scores during treatment. Moreover, the interaction between baseline DFlex-rigidity scores and time was non-significant both during CRT/SAT (*t*(289)=-0.464, *p*=0.643) and TAU (*t*(91.2)=1.51, *p*=0.134), implying that DFlex performance did not moderate changes in the Y-BOCS and EDE-Q scores during treatment.

### Do changes in cognitive flexibility mediate the treatment outcomes?

The mediator models depicted in the right column of **Figure 1** illustrate that in both treatment phases, i.e. CRT/SAT and TAU, there was no significant interaction between the TMT-AB-ratio T0-T1 difference score and time (t(296)=-0.390, *p*=0.697 and (t(112)=0.661, *p*=0.510, respectively). The interaction between the CWIT T0-T1 difference score and time was also not significant for either phase (t(282)=0.641, *p*=0.522, and t(151)=0.882, *p*=0.379, respectively), as was the case for the interaction between the DFlex-rigidity T0-T1 difference score and time (t(286)=-0.924, p=0.356; t(92.4)=0.274, p=0.785, respectively). In summary, we found no mediation effect for any of the three measures of flexibility on changes in disease-specific symptom severity.

## Discussion

### Main findings

This study compared the efficacy of CRT and an active control intervention in improving cognitive flexibility in individuals with OCD or AN. It also explored the connection between baseline flexibility and longer-term outcomes in OCD and AN participants, and whether those showing larger improvements following CRT/SAT achieved better outcomes from TAU.

We observed a time-related effect of both adjunctive treatments on both CWIT-rigidity and DFlex-rigidity but not for TMT AB-ratio in both diagnostic groups. In the absence of any time-related between-group (CRT/SAT or OCD/AN) differences, we conclude that CRT did not enhance cognitive flexibility to a greater degree than SAT in both OCD and AN disproving our first hypothesis that CRT would lead to greater improvements on cognitive flexibility as compared to SAT. We accordingly simplified the moderation and mediation models, excluding diagnosis and type of adjunctive treatment to increase the power of our analyses. Our second hypothesis, suggesting that patients with higher levels of cognitive flexibility would respond more favorably to the treatment, was disproven as the moderator scores failed to predict treatment outcomes. Further, our third hypothesis, proposing that better outcomes during TAU were mediated by improved cognitive flexibility was not supported.

In summary, this study demonstrates that CRT did not improve cognitive flexibility more so than SAT did, nor that the degree of cognitive flexibility had a moderating or mediating effect on the outcomes of OCD and AN treatment.

### CRT as an enhancer of cognitive flexibility

Our findings generally align with previous studies that evaluated CRT for AN, which together provide growing evidence that CRT does not enhance cognitive flexibility nor diminish illness severity more so than an active control condition (57–61, 91).

### Cognitive flexibility as a moderator of treatment outcomes

Our findings regarding the lack of a moderating effect of cognitive flexibility on the 6- and 12 month outcomes of TAU for AN and OCD are in contrast with one previous OCD study and three AN studies (47, 48, 92, 93) but in line with two other OCD studies and one AN study (94–96).

As to the studies reporting contrasting findings, we can say that the recent study by Schubert et al. (92) including 112 patients with OCD did find higher self-reported levels of flexibility at baseline to predict lower levels of OCD symptoms at the conclusion of a specialized CBT-based group therapy. However, a different concept of cognitive flexibility was employed, one that aligns more closely with the theory of Acceptance and Commitment Therapy (ACT), where cognitive flexibility is defined as the capacity to be in contact with the present and act in accordance with long-term goals rather than short-term urges. Moreover, the participants received an inpatient multimodal treatment program without elements specifically targeting cognitive flexibility, while its assessment solely relied on a short self-report questionnaire. Having 21 women with AN complete the WCST at baseline to assess cognitive flexibility, Garrett et al. (48) documented that the more proficient performers had better outcomes after 16 weeks of CBT, as indicated by higher BMI scores. But, unlike our study, Garrett et al. (48) used functional magnetic resonance imaging to evaluate if regional brain activation associated with cognitive flexibility predicted treatment response. Evaluating a group of 82 patients with AN, Dingemans et al. (47) observed that poor baseline cognitive flexibility, as assessed with the TMT and WCST, were associated with greater long-term improvements in ED-related quality of life for those having received CRT compared to the control (TAU only) group. Consistent with our study, though, was that the authors also found no moderating effect for their flexibility measures on disorder-specific outcomes (EDE-Q and BMI). Finally, the study by Harper et al. (93) involving 46 patients with AN showed that those participants who still met the diagnosis at follow-up had shown a poorer performance on the TMT and WCST at baseline compared to peers with a BMI higher than 19 in the last 12 months. In line with our study, Harper et al. (93) found no significant difference in TMT-AB-ratio, only in the TMT-B subtest. The authors used a different outcome-measure (three groups: remaining ill, recently recovered and sustaining recovery) whilst, for AN, we used the EDE-Q as outcome which allows for the possibility to detect more subtle changes.

The contrast in the findings summarized above and ours may then be due to the variety in outcome measures that were used in the AN studies and the lack of uniformity in the assessment of cognitive flexibility. The AN studies finding a moderation effect of cognitive flexibility used weight or BMI or quality of life as outcome measure, while those employing the EDE or EDE-Q found no such effect.

Looking at the two OCD studies documenting results that are in accordance with our findings, (94, 95), we observe that both studies used neuropsychological tests (including the TMT and Stroop) to assess cognitive flexibility. The Oldershaw-study including 71 women with AN showed that baseline cognitive flexibility predicted 7% of posttreatment weight gain, but there was no predictive effect when the EDE-Q outcomes were considered (96).

In sum, that our findings both agree and contrast with previous findings can thus partly be explained by the differences in the measures of symptom severity and cognitive flexibility, and statistical methods, which differences complicate a sound comparison of the various results.

### Cognitive flexibility as a mediator of treatment outcome

We found change in cognitive flexibility during treatment not to predict treatment outcome, which is in line with Schubert et al. (92) who also found no relationship between OCD symptom reductions and an improvement in self-perceived cognitive flexibility, thereby contradicting the earlier findings of Twohig et al. (97) who did note that self-reported cognitive flexibility mediated change in OCD symptoms in their sample. In both studies, a different concept of cognitive flexibility that aligns more closely to ACT was employed.

Consistent with our findings, Oldershaw et al. (96) observed that in their AN sample no measure of change in flexibility correlated with clinical EDE-Q improvement and post-treatment weight. In a recent AN study Duriez et al. (36) did detect that increased cognitive flexibility as measured with the Brixton test (98), mediated the improvement in daily-life functioning as well as ED and depressive symptoms during treatment but not the improvement of BMI. In contrast with our study, Duriez et al. (36) used *t*-tests to detect differences between baseline and follow-up and did not include an adjunctive treatment targeting cognitive flexibility prior to TAU.

Again, these conflicting findings can be explained by the different concepts and measures of cognitive flexibility, different outcome measures, design, and statistical methods. In the case of AN research, it clearly matters whether the EDE-Q is chosen as the outcome measure or weight/BMI.

### Cognitive flexibility - a lack of uniformity in both definition and operationalization

In this study we used two widely recognized neuropsychological tasks and one subjective measure (DFlex) to test cognitive flexibility. We found no correlation between the three baseline flexibility indices in our sample. The concept of cognitive flexibility is a complex one, with a wide variety of tasks and approaches being used to capture its essence (99). Also in the CRT literature, the heterogeneity in the tasks and measures used to operationalize cognitive flexibility is large (61). In their systematic review and meta-analysis, Howlett et al. (100) look at the associations between the two mostly used broad approaches to assess cognitive flexibility: self-report and neuropsychological testing. Self-reporting has the advantage that outcomes are closer to the daily-life experiences of the respondent than those of standardized neuropsychological tasks. However, subjective assessments are susceptible to reporting bias and depend on an individual’s subjective perception of their own abilities, and they, unavoidably, simultaneously gauge other processes of executive functioning. Neuropsychological tests, on the other hand, may not entirely capture all aspects of cognitive flexibility that are important for targeting psychotherapeutic interventions (101). Moreover, both in HCs and patient populations, the relationship between self-report and neuropsychological indices of cognitive flexibility is absent (43, 100), which may be due to task impurities (100, 102) in that any executive task also taps nonexecutive functions that influence the test outcomes just as is the case with self-reported flexibility tools. We hence emphasize that self-report questionnaires cannot be considered valid substitutes of neuropsychological tests of cognitive flexibility and vice versa.

One possibility is that the measures used were insufficiently sensitive to detect meaningful changes over time or failed to capture the complexity of cognitive flexibility and its disorder-specific manifestations. Sample characteristics may also have played a role. Variability in baseline cognitive flexibility, symptom severity, comorbidities, or therapy engagement could obscure potential associations. Additionally, the relatively small sample size may have limited statistical power to detect subtle effects. However, this raises the question of whether neuropsychological measures and self-report questionnaires provide ecologically valid assessments capable of capturing the complexity of cognitive flexibility in real world daily life situations.

Finally, these findings suggest a need to refine our understanding of cognitive flexibility’s role in these disorders. While theoretical models posit that enhanced cognitive flexibility supports treatment responsiveness, the results indicate that this relationship may be more context-dependent, and potentially influenced by factors such as emotional regulation or environmental stressors.

### Limitations

With respect to the limitations of our study, we need to mention the dropout at the 12-month timepoint, which, with 49%, was far higher than expected. Relevantly, 36 of the 48 (75%) of the patients dropping out had already ended TAU and their participation to the study before the 52-week assessment. Relevantly, with 19%, the dropout during the first phase of treatment (at 5 weeks) was more acceptable. Since we calculated the moderators and mediators for the total sample over the full 52-week study period, the chance of non-selective dropout cannot be excluded.

Secondly, three participants (3.3%) with a BMI above 18.5 kg/m² were included, which means that according to DSM-IV criteria, they formally do not fulfill an diagnosis. However, according to the diagnostic procedure of the department where they were treated, they fulfilled an diagnosis.

Thirdly, given that TMT AB-ratio scores following CRT/SAT were non-significantly lower than the baseline scores, we cannot fully rule out that enhancing cognitive flexibility needs more time and/or more training for effects to become manifest. Possibly, a more intensive CRT format [as suggested for individuals with schizophrenia (103)] or a dedicated drill-and-practice strategy throughout the OCD or AN treatment or, finally, apps specifically designed to enhance cognitive flexibility might be more effective in training this executive function. We cannot preclude that in the long run significantly lower scores can be achieved that do contribute to a better treatment outcome.

Finally, a limitation of this study is the absence of data on baseline comorbidities and their potential impact on treatment trajectories. Future research should address these factors to clarify their role in outcomes.

### Final conclusions and further directions

In view of earlier and recent conflicting findings, we question whether CRT should be applied when the aim is to address cognitive inflexibility. It has already been suggested that CRT may not work by resolving this inflexibility but by having patients reflect on their thinking styles and strategies and by translating new behaviors to everyday life. Furthermore, CRT also addresses the common issue of treatment ambivalence in this population, where low motivation to change behaviors is prevalent. CRT tackles this indirectly by providing patients with EDs opportunities to experience treatment successes (104).

We like to suggest several options for future research. First, colleagues should focus on how to define cognitive flexibility, providing an answer as to whether we should use a broad concept or a more precise definition that aligns either closely with existing or novel neuropsychological or with self-report measures. Furthermore, it would be interesting to look for dedicated interventions that directly train cognitive flexibility in OCD and AN, as various apps are targeting this inefficiency.

In AN research, therapy targeting cognitive flexibility, central coherence, and emotional factors, such as CREST (105, 106), is under investigation. While its application in OCD remains unexplored, further study in both AN and OCD populations may be worthwhile. Moreover, the potential of SAT’s focus on behavioral activation (107) and positive psychology, which taps into elements of positive psychology like addressing pleasure, engagement, meaning making (108, 109) warrants further investigation.

The gain in cognitive flexibility from CRT was not great in our study. Based on this and other results, as an integral treatment for all patients with OCD and AN, CRT appears insufficiently effective in improving this ability. We would like to know whether patients with AN or OCD that have a clearer deficit in cognitive flexibility will benefit more from targeted training on this deficit. Finally, CRT research might investigate other mechanisms of action, such as improving treatment ambivalence, the therapeutic relationship, promoting play, reflecting on thinking styles and meaning making.

## Data Availability

The raw data supporting the conclusions of this article will be made available by the authors, without undue reservation.

## Footnotes

1 In the Netherlands, the initial version of the DSM-5 was released in 2014, after participant inclusion for the study had already commenced. The DSM-5 was not fully implemented in the Netherlands until 2017. We chose not to switch DSM versions during the study to maintain consistency.
